# The contribution of a 9p21.3 variant, a *KIF6 *variant, and C-reactive protein to predicting risk of myocardial infarction in a prospective study

**DOI:** 10.1186/1471-2261-11-10

**Published:** 2011-03-15

**Authors:** Dov Shiffman, Ellen S O'Meara, Charles M Rowland, Judy Z Louie, Mary Cushman, Russell P Tracy, James J Devlin, Bruce M Psaty

**Affiliations:** 1 Celera, 1401 Harbor Bay Parkway, Alameda, CA 94502 USA; 2 Group Health Research Institute, Seattle, WA 98101 USA; 3 Department of Medicine and Pathology, University of Vermont, Colchester Research Facility, Colchester VT 05446 USA; 4 Departments of Pathology and Biochemistry, College of Medicine, University of Vermont, Burlington, VT 05405-0068 USA; 5 Departments of Medicine, Epidemiology, and Health Services, University of Washington, Seattle, WA 98115 USA

## Abstract

**Background:**

Genetic risk factors might improve prediction of coronary events. Several variants at chromosome 9p21.3 have been widely reported to be associated with coronary heart disease (CHD) in prospective and case-control studies. A variant of *KIF6 *(719Arg) has also been reported to be associated with increased risk of CHD in large prospective studies, but not in case-control studies. We asked whether the addition of genetic information (the 9p21.3 or *KIF6 *variants) or a well-established non-genetic risk factor (C-reactive protein [CRP]) can improve risk prediction by the Framingham Risk Score (FRS) in the Cardiovascular Health Study (CHS)--a prospective observational study of risk factors for cardiovascular disease among > 5,000 participants aged 65 or older.

**Methods:**

Improvement of risk prediction was assessed by change in the area under the receiver-operator characteristic curve (AUC) and by net reclassification improvement (NRI).

**Results:**

Among white participants the FRS was improved by addition of *KIF6 *719Arg carrier status among men as assessed by the AUC (from 0.581 to 0.596, P = 0.03) but not by NRI (NRI = 0.027, P = 0.32). Adding both CRP and 719Arg carrier status to the FRS improved risk prediction by the AUC (0.608, P = 0.02) and NRI (0.093, P = 0.008) in men, but not women (P ≥ 0.24).

**Conclusions:**

While none of these risk markers individually or in combination improved risk prediction among women, a combination of *KIF6 *719Arg carrier status and CRP levels modestly improved risk prediction among white men; although this improvement is not significant after multiple-testing correction. These observations should be investigated in other prospective studies.

## Background

The Framingham Risk Score (FRS) is a risk prediction model developed by the Framingham investigators to predict the probability of developing coronary heart disease (CHD) [1]. This risk prediction model calculates the probability of a CHD event over a given time period for men and women separately by integrating information about traditional risk factors for CHD, including age, blood pressure, low-density lipoprotein cholesterol (LDL-C), high-density lipoprotein cholesterol (HDL-C), smoking behavior, and diabetes status. The FRS has been evaluated in a number of large population studies and has been shown to predict CHD risk among individuals from different populations and a variety of ethnicities [2]. Because the FRS models were developed as sex-specific scores, the validity of FRS was typically evaluated separately in men and women [2].

Several groups have sought to improve or simplify CHD risk prediction by the FRS [3-6] by developing models that include emerging risk factors. More recently, several studies have investigated whether genetic variants associated with CHD could improve CHD risk prediction models, and much attention has been focused on a well-established genetic risk marker in the 9p21.3 locus (see Palomaki *et al. *[7] for a recent meta analysis). The results of these studies have been mixed. One study found that adding 9p21.3 to a traditional risk factor-based model improved the area under the receiver operator characteristic curve (AUC) as well as patient reclassification [8]. A second study found improvement in reclassification, but not in AUC [9]. And a third study found that 9p21.3 did not improve either AUC or patient reclassification [10]. Another gene variant associated with risk of CHD is a nonsynonymous (Trp719Arg) single nucleotide polymorphism in *KIF6*, which encodes a member of the kinesin superfamily. This *KIF6 *variant (rs20455) was associated with increased risk of CHD in the placebo groups of randomized placebo-controlled clinical trials: the secondary prevention Cholesterol and Recurrent Events (CARE) study and the primary prevention West of Scotland Coronary Prevention Study (WOSCOPS) [11]. This *KIF6 *variant was also associated with CHD in prospective population-based studies: Atherosclerosis Risk in Communities (ARIC) study [12], Cardiovascular Health Study [13], and the Women's Health Study [14]. Interestingly, the *KIF6 *variant was not associated with risk of coronary artery disease in case-control studies [15,16], the explanation for the difference between the results from the prospective studies and the case control studies remains to be determined.

It has been suggested that the genetic contribution to CHD risk diminishes with age [17]. Since the contribution of genetic markers to CHD risk models has typically been assessed in prospective studies of middle-aged individuals, we set out to assess the contribution of genetic markers to CHD risk models in the Cardiovascular Health Study (CHS), a prospective study among Americans aged 65 or older who were followed for the occurrence of cardiovascular events [18]. Since the FRS predicts CHD risk in this population [2], CHS provided the opportunity to ask whether genetic risk markers can improve CHD risk prediction by the FRS among older North Americans. As a point of reference, we also investigated whether C-reactive protein (CRP)--a well established [19] non-genetic risk marker--can improve CHD risk prediction in the same population. Therefore, we report here the contribution of 9p21.3, *KIF6 *719Arg and CRP to CHD risk prediction in CHS.

## Methods

### Cardiovascular Health Study

CHS is a prospective observational study of risk factors for cardiovascular disease in older adults. The recruitment and design methods were previously described [18,20]. Briefly, men and women aged 65 years and older were recruited from random samples of Medicare eligibility lists in 4 US communities (Sacramento County, CA; Washington County, MD; Forsyth County, NC; and Pittsburgh, PA) and from age-eligible participants in the same household. Potential participants were excluded if they were institutionalized, not ambulatory at home, under hospice care, receiving radiation or chemotherapy for cancer, not expected to remain in the area for at least 3 years, or unable to be interviewed. CHS enrolled 5201 participants from 1989 to 1990; an additional 687 African American participants entered the cohort from 1992 to 1993. The CHS cohort of 5888 was 57.6% female and 15.7% African American. The mean age at enrollment was 72.8 years (standard deviation 5.6). The institutional review board at each site approved the study methods, and all participants gave written informed consent. Collection of baseline demographic, clinical, and genetic data was previously described [13,18,21,22]. Briefly, participants completed a baseline clinic examination [18] that included a medical history interview, physical examination, and blood draw[21]. Baseline self-reports of MI or stroke were confirmed by information from the clinic examination or by review of medical records or physician questionnaires [22]. Genotypes of the CHS participants were determined using a multiplex method that combines polymerase chain reaction (PCR), allele-specific oligonucleotide ligation assays, and hybridization to oligonucleotides coupled to Luminex 100TM ×MAP microspheres (Luminex, Austin, TX) as previously described [13]. Cardiovascular events during follow-up were identified at semiannual contacts, which alternated between clinic visits and telephone calls. Suspected events were adjudicated according to standard criteria by a physician review panel using information from medical records and, in some cases, interviews with the physician, participant, or a proxy informant [23]. Medicare utilization files were searched to ascertain events that may have been missed. In this analysis, MI was defined as definite or probable nonfatal MI or definite fatal MI.

### Statistical analysis

Participants with unavailable DNA or who did not consent to the use of their DNA for studies by private companies (N = 514) were excluded from the present study. Participants for whom DNA samples were inadequate (N = 130) were also excluded. Analyses also excluded participants who had a baseline history of MI (N = 517 of the 5244 participants), or stroke (N = 222), who were neither white nor African American (N = 30), or who had missing data for calculating the FRS or the three risk markers (n = 284). After applying these overlapping exclusions, 4284 participants remained for analysis.

We conducted analyses of time to incident MI. Follow-up began at CHS enrollment and ended on the date of incident MI, death, loss to follow-up, or June 30, 2006, whichever occurred first. The median time at risk was 12.6 years. Hazard ratios (HRs) and 95% confidence intervals (CIs) were estimated using Cox regression models. Based on their use in the FRS, variables were coded as continuous (age, CRP), or categorical (smoking status, hypertension, LDL-C category, HDL-C category, diabetes, *KIF6 *719Arg carrier status), or ordinal (9p21.3 SNP: rs10757274).

The predicted 10-year risk of incident MI for each participant in this study was calculated based on estimates of the baseline hazard function and the regression coefficients of Cox models that included the FRS variables with or without additional risk markers as described [1]. These analyses were conducted in men and women separately as described [1,2]. The risk markers that were added to the FRS were coded continuously (CRP), additively (9p21.3 SNP), or dominantly (*KIF6 *719Arg) because this coding was consistent with what was reported previously for these risk markers. The change in the AUC was used to assess the improvement of the FRS by additional risk markers.

To assess whether adding risk markers to FRS resulted in reclassification of individuals to more appropriate risk categories, we used the net reclassification improvement (NRI) measure [24]. Briefly, among those with incident events, classification is more appropriate if the individual is reclassified to a higher risk category and less appropriate if the individual is reclassified to a lower risk category. For those without incident events, the converse is true. The difference in the proportion of individuals moving to more appropriate and less appropriate categories is calculated separately for those with and without incident events. The NRI is the sum of these two differences. The predicted CHD probabilities were grouped into 10-year risk categories of 0% to < 5%, 5% to < 10%, 10% to < 20%, and 20% or greater based on FRS models with and without additional risk markers. Statistical tests were two-sided. Data were analyzed using Stata statistical software [25].

## Results

The baseline characteristics that were used to calculate the FRS among the 3651 white participants in this analysis are presented in Table 1, which also presents coefficients from the FRS model. We calculated the AUC for the FRS, using the coefficients estimated among men and women separately because the FRS coefficients were originally derived separately in men and women and because cardiovascular risk is generally different between men and women--men generally have CHD events earlier in life. The AUC among whites was 0.581 for men and 0.619 for women. After adjustment for the FRS risk factors, the risk allele (G) of the 9p21.3 SNP (rs10757274) was associated with increased risk of MI among white men (HR = 1.22; 95%CI 1.03 to 1.45, P = 0.02), this association did not reach statistical significance among white women (HR = 1.16; 95%CI 0.98 to 1.37, P = 0.08). Carriers of one or two 719Arg alleles of *KIF6 *were at increased risk of MI among white men (HR = 1.42; 95%CI 1.11 to 1.82, P = 0.006) but not women (HR = 1.05; 95%CI = 0.83 to 1.33, P = 0.68). Higher CRP was associated with increased risk of incident MI among both white men (HR = 1.28 for 1 standard deviation higher CRP; 95%CI 1.14 to 1.45, P = 0.00005) and white women (HR = 1.15; 95%CI 1.01 to 1.31, P = 0.04).

**Table 1:** Framingham Risk Score Characteristics at Baseline Among White Participants

	Men n = 1495			Women n = 2156	
			
	Baseline Value	β (P value)*		Baseline Value	β (P value)*
Age, y, mean (SD)	73.3 (5.7)	0.043 (0.0002)		72.3 (5.4)	0.18 (0.45)
Current smoker	10%	-0.19 (0.44)		12%	0.49 (0.01)
LDL, mg/dL, mean (SD)	124 (33)	NA§		135 (37)	NA§
LDL category					
< 100	24.4%	-0.13 (0.45)		16.8%	-0.18 (0.38)
100-129	35.0%	reference		30.7%	reference
130-159	28.0%	0.10 (0.49)		29.4%	-0.04 (0.80)
160-189	10.0%	0.22 (0.29)		16.2%	0.10 (0.57)
> = 190	2.7%	-0.19 (0.65)		6.9%	-0.23 (0.41)
HDL, mg/dL, mean (SD)	48 (13)	NA§		59 (16)	NA§
HDL category					
< 35	11.4%	0.16 (0.47)		1.9%	-0.24 (0.65)
35-44	34.1%	0.090 (0.60)		14.2%	0.15 (0.43)
45-49	18.3%	reference		13.0%	0.20 (0.30)
50-59	20.5%	-0.21 (0.29)		26.9%	reference
> = 60	15.6%	-0.030 (0.89)		43.9%	-0.18 (0.27)
Blood pressure category†					
Optimal	23.3%	-0.31 (0.14)		23.8%	0.22 (0.34)
Normal	18.5%	reference		18.0%	reference
high normal	20.2%	0.30 (0.13)		19.2%	0.46 (0.05)
HTN stage I	23.8%	0.39 (0.04)		26.2%	0.58 (0.007)
HTN stage II-IV	14.1%	0.21 (0.33)		12.8%	0.95 ( 0.00005)
Diabetes‡	13.0%	0.57 (0.0006)		8%	0.66 (0.001)

* Coefficients from Cox regression model of Framingham Risk Score (FRS) variables.

† Optimal (systolic < 120 mm Hg and diastolic < 80 mm Hg), normal blood pressure (systolic 120 to 129 mm Hg or diastolic 80 to 84 mm Hg), high normal blood pressure (systolic 130 to 139 mm Hg or diastolic 85 to 89 mm Hg), hypertension stage I (systolic 140 to 159 mm Hg or diastolic 90 to 99 mm Hg), and hypertension stage II-IV (systolic 160 or diastolic 100 mm Hg). When systolic and diastolic pressures fell into different categories, the higher category was selected. Blood pressure categorization was made without regard to the use of antihypertensive medication.

‡ Fasting serum glucose ≥140 or taking insulin or oral hypoglycemic medications.

§ Continuous cholesterol measures are not included in the FRS model

Among white participants, adding rs10757274 in the 9p21.3 locus to the FRS variables, did not improve the AUC among either men or women (P ≥ 0.35, Tables 2 and 3). In the net reclassification improvement (NRI) analysis, adding 9p21 to the FRS reclassified 13.7% of white men and 10.3% of white women to risk categories that were different from the FRS categories. However, the net number of individuals that were classified to more appropriate risk categories was ≤ 25, with the NRI ≤ 0.02 (P ≥ 0.39). Similar results were observed after CRP was added to FRS. The AUC was not improved for either white men or women (P ≥ 0.15) nor was the NRI significant (NRI ≤ -0.01, P ≥ 0.19). The net number of correctly reclassified individuals was ≤ 10 in each of the two sexes. Similarly, adding *KIF6 *719Arg carrier status to the FRS variables did not improve risk prediction by the NRI among either men or women (NRI ≤ 0.027, P ≥ 0.32, the net number of correctly reclassified individuals was ≤ 33). However, adding *KIF6 *719Arg carrier status to the FRS modestly improved the AUC among men (from 0.581 to 0.596; P = 0.03), though not among women (P = 0.57).

**Table 2:** Addition of Risk Markers to the FRS: Effect on Risk Prediction in White Men

Markers Added to FRS Model	Number Reclassified (%)	Net Number Reclassified Correctly (NRI, P value)		AUC (P value)*
9p21.3	205 (13.7)	25	(0.021, 0.41)	0.586 (0.35)
KIF6	309 (20.7)	33	(0.027, 0.32)	0.596 (0.03)
CRP	346 (23.1)	10	(-0.017, 0.61)	0.595 (0.15)
CRP + 9p21.3	412 (27.6)	56	(0.029, 0.40)	0.599 (0.09)
CRP + KIF6	466 (31.2)	86	(0.093, 0.008)	0.608 (0.02)
9p21.3 + KIF6	348 (23.3)	40	(0.041, 0.18)	0.600 (0.03)
CRP + 9p21.3 + KIF6	495 (33.1)	93	(0.076, 0.04)	0.612 (0.01)

* P value for change in the AUC from the AUC of the FRS model (0.581)

**Table 3:** Addition of Risk Markers to the FRS: Effect on Risk Prediction in White Women

Markers Added to FRS Model	Number Reclassified (%)	Net Number Reclassified Correctly (NRI, P value)		AUC (P value)*
9p21.3	222 (10.3)	-16	(-0.018, 0.39)	0.621 (0.57)
KIF6	72 (3.3)	0	(0.007, 0.61)	0.618 (0.57)
CRP	270 (12.5)	-10	(-0.032, 0.19)	0.617 (0.69)
CRP + 9p21.3	362 (16.8)	20	(0.007, 0.81)	0.620 (0.81)
CRP + KIF6	276 (12.8)	-4	(-0.029, 0.24)	0.617 (0.67)
9p21.3 + KIF6	246 (11.4)	-2	(0.015, 0.52)	0.620 (0.65)
CRP + 9p21.3 + KIF6	357 (16.6)	3	(-0.002, 0.95)	0.620 (0.81)

* P value for change in the AUC from the AUC of the FRS model (0.619)

**Figure 1:**
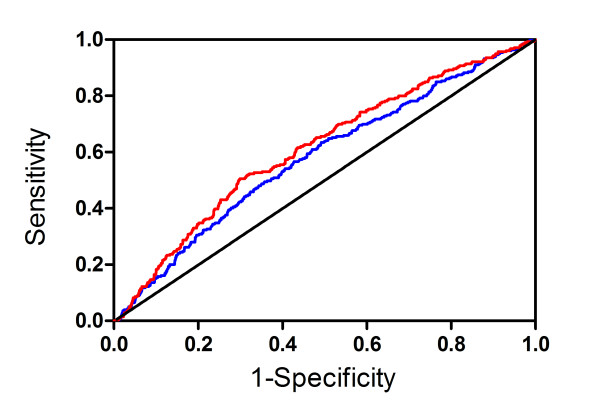
**Risk Prediction among White Men in the Cardiovascular Health Study**. Receiver operator characteristic curves calculated for the white men participants of CHS. Blue: Framingham risk score. Red: *KIF6*, 9p21.3, and CRP added to the Framingham risk score.

Since the nominal value of the AUC after addition of an individual risk marker to the FRS was larger than the AUC of FRS alone (Tables 2 and 3), we asked if adding more than one marker to the FRS further improved risk prediction. Adding all three markers to the FRS resulted in the largest AUC among white men (0.612, P = 0.01 for difference from the FRS AUC, Figure 1) and largest net number of correctly reclassified individuals (93, P = 0.04). However, adding all three markers to the FRS did not improve risk prediction among white women. Adding both CRP and 9p21.3 to the FRS, resulted in a marginal increase in the AUC among white men (to 0.599; P = 0.09), but not among white women. The NRI did not indicate improvement of risk prediction among either white men or women (P ≥ 0.4). In contrast, addition of *KIF6 *with either CRP or 9p21.3 to the FRS resulted in a larger AUC among white men (0.600 and 0.608 respectively; P ≤ 0.03) but not among women. The combination of *KIF6 *and CRP also improved risk prediction by the NRI among white men (NRI = 0.093, P = 0.008, the net number of correctly reclassified individuals = 86) but not among women. However, the NRI was not significant when men and women were combined (NRI = 0.026, P = 0.2).

For the 228 African American men and 405 women (Table 4), none of the 3 risk markers were associated with incident MI (P ≥ 0.05). The power to detect association between the genetic risk factors and MI was < 37% to detect a risk ratio of 1.5. Thus, all results among African Americans are reported in the online supplement. Among African American participants of CHS, only 9p21.3 improved risk prediction among men as assessed by NRI (NRI = 0.182; P = 0.02, net number of correctly reclassified individuals = 10). None of the other markers individually or in combination improved risk prediction by the FRS (Tables 5 and 6).

**Table 4:** Framingham Risk Score Characteristics at Baseline among Black Participants

	Men n = 228			Women n = 405	
			
	Baseline Value	β (P value)*		Baseline Value	β (P value)*
Age, y, mean (SD)	72.3 (5.5)	0.035 (0.33)		73.0 (5.6)	1.37 (0.05)
Current smoker	23%	0.28 (0.55)		14%	0.0085 (0.99)
LDL, mg/dL, mean (SD)	124 (32)	NA§		132 (37)	NA§
LDL category					
< 100	22.4%	0.36 (0.46)		17.8%	1.08 (0.02)
100-129	34.2%	reference		33.3%	reference
130-159	31.6%	-0.34 (0.51)		29.4%	0.67 (0.13)
160-189	9.6%	0.92 (0.11)		12.6%	0.52 (0.33)
> = 190	2.2%	0.066 (0.95)		6.9%	1.20 (0.02)
HDL, mg/dL, mean (SD)	53 (14)			61 (15)	
HDL category					
< 35	4.4%	-0.22 (0.84)		0.7%	
35-44	23.2%	-0.75 (0.15)		11.85	-0.43 (0.45)
45-49	21.5%	reference		9.9%	-0.18 (0.76)
50-59	24.1%	-1.06 (0.05)		29.9%	reference
> = 60	26.8%	-0.69 (0.16)		47.6%	-0.17 (0.64)
Blood pressure category†					
Optimal	17.1%	-0.46 (0.52)		13.6%	0.59 (0.50)
Normal	17.5%	reference		14.1%	reference
high normal	19.3%	0.080 (0.89)		18.0%	0.81 (0.32)
HTN stage I	32.0%	0.34 (0.52)		31.1%	1.01 (0.18)
HTN stage II-IV	14.0%	0.67 (0.29)		23.2%	1.55 (0.04)
Diabetes‡	19%	-0.15 (0.76)		20%	0.51 (0.17)

* Coefficients from Cox regression model of Framingham Risk Score (FRS) variables.

† Optimal (systolic < 120 mm Hg and diastolic < 80 mm Hg), normal blood pressure (systolic 120 to 129 mm Hg or diastolic 80 to 84 mm Hg), high normal blood pressure (systolic 130 to 139 mm Hg or diastolic 85 to 89 mm Hg), hypertension stage I (systolic 140 to 159 mm Hg or diastolic 90 to 99 mm Hg), and hypertension stage II-IV (systolic 160 or diastolic 100 mm Hg). When systolic and diastolic pressures fell into different categories, the higher category was selected. Blood pressure categorization was made without regard to the use of antihypertensive medication.

‡ Fasting serum glucose ≥140 or taking insulin or oral hypoglycemic medications.

§ Continuous cholesterol measures are not included in the FRS model

**Table 5:** Addition of Risk Markers to the FRS: Effect on Risk Prediction in Black Men

Markers Added to FRS Model	Number Reclassified (%)	Net Number Reclassified Correctly (NRI, P value)	AUC (P value)*
9p21	50 (21.9)	10 (0.182, 0.02)	0.688 (0.07)
KIF6	no data†	no data†	no data†
CRP	48 (21.1)	-4 (0.006, 0.94)	0.654 (0.92)
CRP + 9p21	70 (30.7)	10 (0.129, 0.16)	0.675 (0.51)
CRP + KIF6	no data†	no data†	no data†
9p21 + KIF6	no data†	no data†	no data†
CRP + 9p21 + KIF6	no data†	no data†	no data†

* P value for change in AUC from AUC of the FRS model (0.657)

†Risk for KIF6 cannot be calculated because none of the 15 African American men who were noncarriers of the KIF6 719Arg risk allele had an event.

**Table 6:** Addition of Risk Markers to the FRS: Effect on Risk Prediction in Black Women

Markers Added to FRS Model	Number Reclassified (%)	Net Number Reclassified Correctly (NRI, P value)	AUC (P value)*
9p21	110 (27.2)	12 (0.013, 0.89)	0.734 (0.40)
KIF6	11 (2.7)	-1 (-0.023, 0.35)	0.723 (0.94)
CRP	31 (7.7)	13 (0.016, 0.70)	0.726 (0.63)
CRP + 9p21	118 (29.1)	14 (0.039, 0.70)	0.735 (0.39)
CRP + KIF6	33 (8.1)	9 (0.025, 0.60)	0.725 (0.83)
9p21 + KIF6	113 (27.9)	15 (0.042, 0.66)	0.733 (0.46)
CRP + 9p21 + KIF6	119 (29.3)	15 (0.002,0.99)	0.734 (0.46)

* P value for change in AUC from AUC of the FRS model (0.723)

## Discussion

We investigated whether adding a 9p21.3 variant, a *KIF6 *variant, or CRP to the FRS could improve MI risk prediction in CHS, a large prospective study of individuals aged 65 years or older from American communities. Adding individual risk markers had, at best, a modest effect on risk prediction. Adding two or more risk markers, specifically risk marker combinations that included the *KIF6 *719Arg genotype, resulted in a somewhat higher, though still modest improvement in risk prediction as measured among white males by either the AUC or by NRI, but not in the combined male and female population.

The appropriate methods for assessing improvement of risk prediction by risk markers have been widely debated [26]. And given the limitations of the AUC measure, alternative assessment methods such as NRI have been proposed [24]. Although the current study was not designed to compare the AUC and NRI, the results from these two measures were largely consistent.

The ability of CRP to improve risk stratification has been evaluated in multiple studies, which found that the addition of CRP to risk prediction models provided modest improvement in risk prediction (see Buckley *et al. *[19] and Schnell-Inderst *et al. *[27] for recent comprehensive evaluations). In CHS, CRP did not improve risk prediction by the FRS as assessed by the AUC or NRI measures, consistent with previously published observations that the association of a risk factor with MI after adjustment for the FRS variables does not necessarily mean that the risk factor will improve risk prediction beyond the FRS, as measured by the change in the AUC, partly because even risk factors with large odds ratio (up to 7) have distributions that overlap substantially between those with and without disease [28,29]. However, the addition of CRP in combination with *KIF6 *719Arg to the FRS improved risk prediction by both the AUC and NRI measures among white men.

Although the association between CHD and SNPs in the 9p21.3 locus has been reported extensively [7], Dehghan *et al. *[30] reported that SNPs in this locus were not associated with risk of CHD in a prospective study of older Europeans. In contrast, we have observed in CHS, a population of individuals 65 years and older, that a SNP in the 9p21.3 locus is associated with MI among men, although this association did not reach statistical significance among women. We have also observed in CHS an association between MI and *KIF6 *719Arg carrier status among men but not among women. However, published results from the Women's Health Study [10,14] suggest that both 9p21.3 and *KIF6 *719Arg are associated with MI among women. These inconsistent findings among women could be due to lack of power in CHS, or to different baseline risk of MI in CHS and the Women's Health Study. Additional analysis of the 9p21.3 and *KIF6 *variants in prospective studies of women would be required to understand this apparent inconsistency.

The addition of the 9p21.3 variant to the FRS did not improve risk prediction in CHS as measured by either the AUC or NRI among whites. Similarly, addition of a SNP in the 9p21.3 locus did not improve risk prediction by traditional risk factors in the Women's Health Study [10]. However, in ARIC [8] the addition of 9p21.3 to the FRS resulted in a modest but statistically significant improvement in risk prediction as measured by AUC or reclassification, and in the Northwick Park Heart Study II [9] adding 9p21.3 improved reclassification but not AUC. These inconsistent results could be attributed to differences in baseline risk between the studies, (the older CHS population is likely to have greater baseline risk of MI), which could affect the power to detect the modest improvement in risk prediction contributed by the 9p21.3 variant.

Genetic and non-genetic biomarkers offer different benefits in the assessment of CHD risk. Non-genetic biomarkers could change over time, and therefore, repeat measurements may be necessary because of day-to-day variation in the level of these biomarkers. However, repeat measurements of non-genetic biomarkers may also provide an indication of successful medical therapy or life-style modification. Genetic biomarkers do not change and thus need only be measured once to obtain information about the lifelong exposure to that biomarker. The 9p21.3 and *KIF6 *gene variants were chosen for investigation because they have both been reported to be associated with CHD in multiple prospective studies and are common variants. For example, in the white population about 75% of carry at least one risk allele of 9p21.3 and about 65% carry at least one *KIF6 *719Arg risk allele. CRP was chosen because of the well-established association between CRP levels and risk of CHD and because of continuing interest in whether it should be added to risk prediction algorithms. Elevated CRP is also common. For example, although CRP was analyzed as a continuous variable in this study in order to increase the power of the study, others have reported that in CHS, 26% of the population have elevated CRP (> 3 mg/dL) [31]. Thus, ~17% of the CHS white population have both elevated CRP and carry the 719Arg allele of *KIF6*.

This study has several limitations. The AUC of the FRS model for white men (0.581) and white women (0.619) in this study of older individuals is lower than the AUC that has been reported for middle age populations (e.g., 0.75 and 0.83 among white men and among white women in ARIC [2]), thus the markers we studied may only improve risk prediction in populations in which the ability of the FRS to predict CHD is modest. The difference observed between the AUC and NRI measures for the addition of *KIF6 *719Arg among men could be attributed to the overall modest improvement of risk prediction by single marker addition. Another limitation of this study is the limited number of genetic markers evaluated--a recent paper suggested that risk prediction models might be improved by incorporating large number of genetic markers into a genetic risk score [32]. Lastly, the effect of these single and multiple marker additions on risk prediction was investigated in a population of individuals aged 65 or older at baseline and our observations may not be generalizable to younger populations.

## Conclusions

In the white male population of CHS, the addition of *KIF6 *719Arg in combination with 9p21.3, CRP, or both modestly improved risk prediction. This improvement was not significant after multiple-testing correction and was not observed in the combined male and female population.

## Conflict of Interest

DS, CMR, JZL, and JJD are employees of Celera

## Sources of Funding

The research reported in this article was supported by contracts N01-HC-15103, N01-HC-35129, N01-HC-45133, N01-HC-55222, N01-HC-75150, N01-HC-85079 through N01-HC-85086, and U01 HL080295 from the National Heart, Lung, and Blood Institute, with additional contribution from the National Institute of Neurological Disorders and Stroke. R.P.T. was supported by NIH RO1 HL077499

## Authors' contributions

Study design: DS, MC, RPT, JJD, BMP. Data collection: JZL, MC, BMP. Data analysis: ESO, JZL, CMR. Manuscript writing: DS, ESO, CMR, MC, RPT, JJD, BMP. All authors read and approved the final manuscript.

## Pre-publication history

The pre-publication history for this paper can be accessed here:

http://www.biomedcentral.com/1471-2261/11/10/prepub
